# Toward Linking Indoor Commercial Source Emissions to Outdoor Volatile Organic Compounds Using Mobile Measurements

**DOI:** 10.1021/acsestair.5c00290

**Published:** 2026-04-22

**Authors:** Sri Hapsari Budisulistiorini, Thomas C. Moore, Marvin D. Shaw, Will S. Drysdale, James D. Lee, David C. Carslaw

**Affiliations:** Wolfson Atmospheric Chemistry Laboratories, Department of Chemistry, 8748 University of York, York YO10 5DD, U.K.

**Keywords:** indoor emissions, volatile organic compounds (VOCs), mobile monitoring, Gaussian plume modeling, spatial modeling, urban air quality

## Abstract

Assessing the impact of indoor volatile organic compound (VOC) sources on outdoor concentrations remains challenging due to their variability, rapid dispersion, and chemical reactions in the atmosphere. Mobile monitoring can address these challenges by providing spatial and temporal resolution of localized emission sources. In this study, we developed a new approach to characterize the impact of indoor emissions on outdoor air quality using mobile measurements. We used geographic information to identify the locations of hundreds of individual source types in Bradford, England, including restaurants, beauty salons, and automobile repair shops. For each source type, we modeled the potential hourly contribution using an advanced Gaussian plume model—incorporating wind speed and direction—across 22 mobile measurement circuits (approximately 56 modeled hours). The outcome is a single *source factor* for each latitude-longitude coordinate at each hour of the measurement campaign, representing the influence level of each source type. We then applied K-means clustering to group *source factors* based on their spatial distributions and influence levels, and analyzed their relationship with the incremental concentrations of VOCs and NO_*x*_ using bootstrap-validated generalized additive models (GAMs). Several previously identified key tracer compounds showed robust associations with specific *source factors*, including *m*/*z* 102 (tentatively assigned as butanone) with *auto repair source factor*, *m*/*z* 59 (acetone) with *beauty salon source factor*, and *m*/*z* 68 (isobaric compounds: isoprene and furan) with *restaurant source factor*. This method offers a new perspective on air quality monitoring by using source location information to inform the analysis of mobile measurements, providing a robust framework for identifying the outdoor signatures of commercial indoor activities.

## Graphical abstract

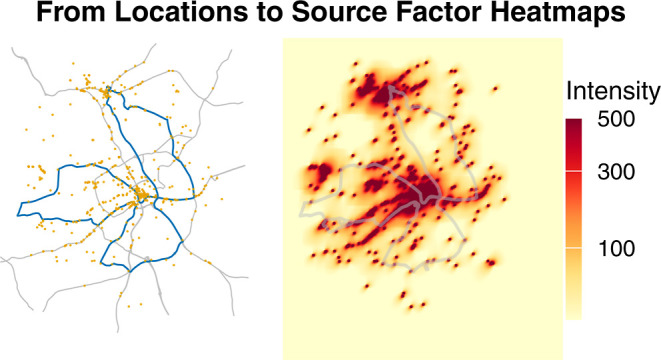

## Introduction

Indoor environments encounter significant pollution from volatile organic compounds (VOCs) from building materials, furniture, and occupant activities, including cooking^1^ and the use of personal care products (PCPs).^2^ These emissions are also affected by ventilation rates and atmospheric chemical reactions.^3^ Exposure to VOCs indoors has been linked to adverse health effects, such as sick-building syndrome and respiratory symptoms.^4^ These VOC emissions can also escape into the urban atmosphere, where reductions in vehicle tailpipe emissions have made them more prominent.^2^ However, assessing the impact of indoor VOC sources on outdoor air quality remains challenging due to their variability, rapid dispersion, and complex atmospheric reactions.

While fixed monitoring stations provide valuable air quality data for major pollutants, they lack the spatial and temporal resolution to identify specific emission sources. Mobile measurement techniques have emerged as a valuable tool offering rapid, spatial, and temporal pollutant measurements. Advances in mobile air quality monitoring have significantly improved the spatiotemporal resolution of urban measurement data for air pollutants, such as nitrogen oxides (NO_*x*_),^5^ VOCs,^6^ and speciated particulate matter (PM).^7^ Mobile methods have also been instrumental in assessing non-tailpipe emissions of VOCs^8^ and cooking emissions^9^ on urban streets. However, most of these studies focused on monitoring a limited set of gases and particles associated with specific sources rather than the complex mixtures of species emitted from diverse sources.

Characterization of air pollutant sources is commonly achieved using receptor models, including chemical mass balance models and multivariate techniques such as positive matrix factorization and principal component analysis.^10–12^ Source profiling, which relies on unique chemical fingerprints of emissions from known activities, is also widely applied for sources such as nonexhaust emissions,^13^ biomass burning emissions,^14^ and cooking emissions.^15^ While these approaches have proven highly effective, they often require either detailed, high-resolution chemical speciation data or well-constrained source profiles. Moreover, overlapping chemical signatures across sources can complicate interpretation, particularly in environments where multiple sources coexist and emit similar compounds.

Indoor environments are characterized by numerous VOCs from coexisting and often colocated sources. Acetone is abundant in beauty salons^16^ due to emissions from hair care and body products, and fragrances, while also being used in spray coatings for automobile repair shops.^17^ Other important VOC groups measured indoors include esters (e.g., ethyl acetate), aromatics (e.g., toluene, xylene, benzene), terpenes (e.g., isoprene, monoterpenes), aldehydes (e.g., acetaldehyde), alcohols (e.g., ethanol), and volatile methyl siloxanes (VMS).^18,19^ VMS compounds such as octamethylcyclotetrasiloxane (D4) and decamethylcyclopentasiloxane (D5) are widely observed in homes, supermarkets, schools, and offices due to the use of PCPs,^20–22^ and are also detected in urban air from vehicle-related applications.^8^ Residential cooking further contributes to VMS and low-volatility siloxane emissions through the use of silicone baking molds^23^ and polymeric oven interiors.^24,25^

Additional tracers, such as nonanal, highlight the complexity of indoor-related emissions. Nonanal is produced from the oxidation of oleic acid in cooking oil emissions,^26,27^ with an average concentration of 370 parts per trillion (ppt) reported in kitchens,^28^ but it is also emitted from building materials,^29^ human skin,^30^ meat products,^31,32^ and rice.^33^ Similarly, benzene, toluene, and xylene, commonly associated with vehicle exhaust,^34^ are also detected in car showrooms and automobile repair shops,^17,35^ and restaurant exhausts.^36^ This extensive overlap in chemical composition across source types, combined with oxidation processes and dispersion, underlines the limitations of relying solely on exact chemical fingerprints for source attribution.

In this study, we introduce a new approach for linking emission sources to air pollutants that focuses on incremental changes and covariation, rather than absolute concentrations. Using fast-response mobile measurements collected across the city of Bradford, England, we examine how VOC levels systematically increase with the local density and proximity of specific source types. Source attribution is approached from the perspective of spatial source information, using the known locations of hundreds of individual sources—including restaurants, beauty salons, and automobile repair shops (hereafter referred to as auto repair shops)—without directly measuring the chemical fingerprints or magnitude of each emission source.

We develop a *source factor* metric that quantifies the cumulative influence of nearby sources by accounting for source proximity and dispersion, and relates this metric to observed increases in pollutant levels. This method leverages consistent spatial trends in pollutant responses to increasing source density. By integrating geographic source data with speciated mobile measurements and existing knowledge of source profiles, this approach provides an independent and complementary line of evidence to established source apportionment models. The *source factor* framework is flexible, scalable, and transferable to other urban environments, enabling the investigation of both established and emerging emission sources using widely available spatial information (e.g., Google Maps) without requiring a priori knowledge about source-pollutant relationships.

## Methods

### Mobile Measurements and Instrumentation

We conducted air quality measurements in Bradford, a metropolitan city in northern England with a total population of 560,200 in 2024, as shown in Figure 1. This study is part of the INGENIOUS project^37^ built on the Born In Bradford (BiB) study.^38^ The measurement campaign spanned two periods: February–March and June–July of 2023, during different times of day: morning (10:00–12:00), afternoon (13:00–15:00), and evening (16:00–18:00) local time, and both weekdays and weekends. We conducted 22 repeated measurement circuits totaling 127,371 s, or approximately 35 h of mobile time series, corresponding to approximately 56 modeled hours (see Emission Source Factor section). The mobile measurements were conducted on a fixed route as indicated in Figure 1. The route was designed to cover Bradford and neighboring towns and villages, covering a range of population and business densities, and potential indoor source emissions.

**Figure 1:**
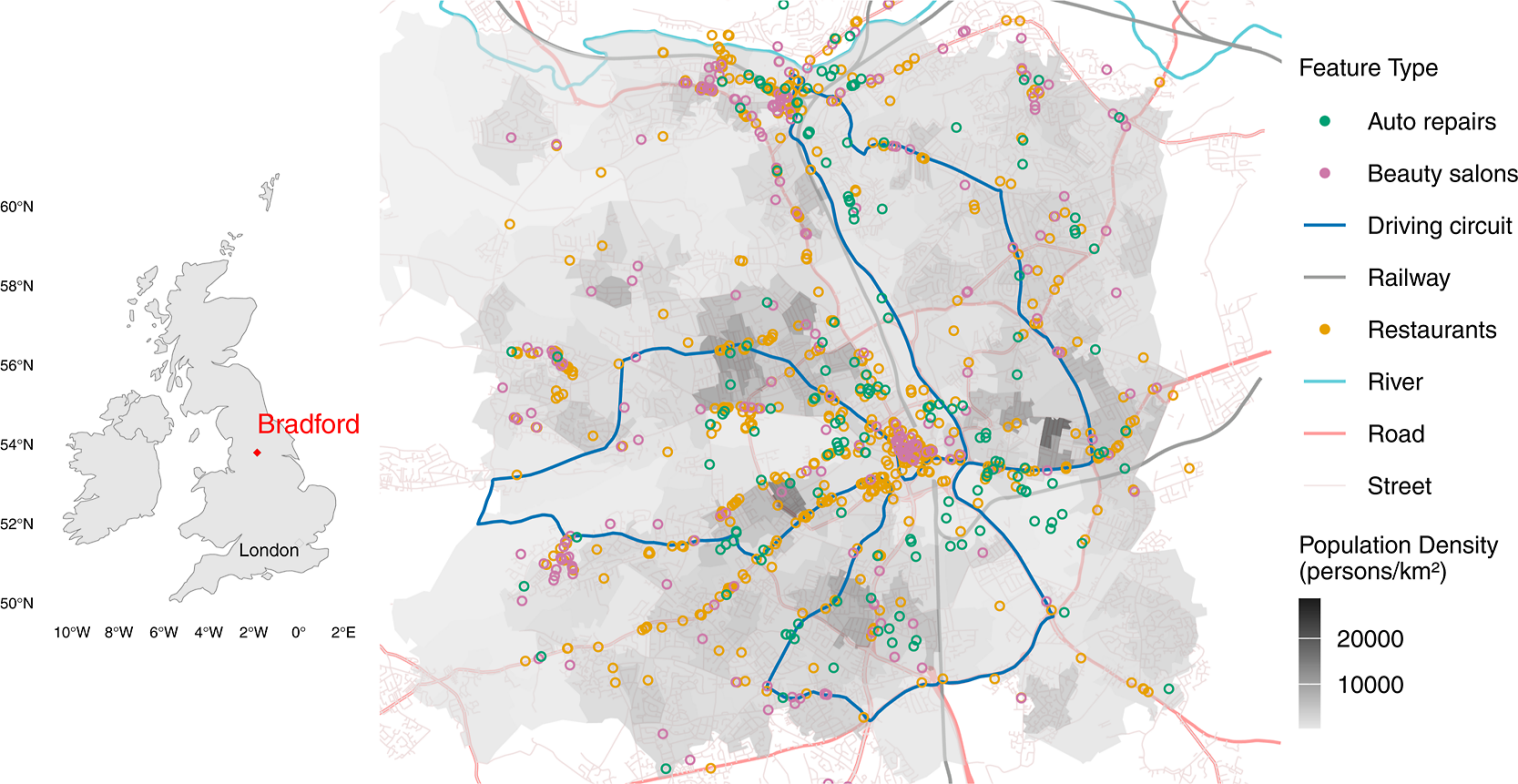
Study area, the City of Bradford, is shown on the map of the British Isles (left panel) and in greater detail on the right panel. The colored areas on the detailed map represent Bradford’s population density, estimated from the Lower-layer Super Output Area (LSOA, Office of National Statistics). Key features, including railways, rivers, roads, and streets, were sourced from OpenStreetMap. The measurement driving circuit, designed specifically for this study, traverses the city to capture a range of activities and population density variations.

The mobile measurements were performed using an instrumented mobile laboratory, the Wolfson Atmospheric Chemistry Laboratories Air Sampling Platform (WASP), described in detail in previous studies.^5,6^ Briefly, the WASP samples air from a front-facing inlet near the driver’s side at 2.25 m above the ground. Previous tests have indicated minimal occurrences of self-sampling from the exhaust, and we excluded measurements when the van was parked or reversing to minimize the instances of self-sampling. Two 12VDC 230 Ah batteries power the instruments. A Garmin GPS 18x PC, externally mounted at a height of 2.5 m, measures geographical locations, speed over ground, and vehicle direction. A customized DAQFactory program collects data measured by various instruments.

#### SIFT-MS

The WASP is equipped with a selected-ion flow-tube mass spectrometer (SIFT-MS, Voice200 Ultra, Syft Technologies, New Zealand) for measuring VOCs and inorganic gases. Detailed operational principles of the SIFT-MS are available elsewhere,^39^ but a brief description is provided here.

The SIFT-MS features a custom-built multiport sample inlet capable of autonomously selecting between sample, zero, and calibrant gases via the control software (Labsyft 1.6) and employs fast reagent ion switching among H_3_O^+^, NO^+^, and O_2_^+^ for each mass. These ions are generated from air and water in a microwave plasma ion source at around 460 mTorr, with a nitrogen carrier gas (Research grade, BOC) supplied at 0.6 Torr L s^–1^ and a sample flow rate of 100 standard cm^3^ min^–1^.

To maximize spatial data density during mobile measurements, the acquisition rate was optimized to 100 ms ion dwell time to scan 72 masses, resulting in a total measurement time of 7.2 s per cycle. The mass list includes replicate scans of certain masses using different reagent ions, while others (e.g., mass-to-charge ratios (*m*/*z*) 45) were scanned using only a single reagent ion. For the final data set, one product ion was selected for each compound, resulting in a total of 35 species (34 VOCs and hydrogen sulfide H_2_S) representative of emission tracers from indoor activities such as cooking, cleaning, and PCPs, provided in Table S1 of the Supporting Information.

The mixing ratio of each compound was determined from the measured product ion signals generated by ionization with reagent ions. Reagent ions are extracted into a quadrupole chamber at approximately 5 × 10^–4^ Torr with a 10 L s^–1^ turbo-molecular pump, then passed through electrostatic lenses and a mass filter. Ions that are not rejected are thermalized in nitrogen before selectively ionizing the target mass. The resulting product ions pass through a downstream quadrupole mass filter to a secondary electron multiplier detector, where they are separated by their *m*/*z* and counted.

Analyte mixing ratios, reported in parts per billion (ppb), are calculated based on ion–molecule reactions as follows^6^

1 $$\left[A\right]=\gamma \times \frac{\left[P^{+}\right]}{\left[R^{+}\right]t_{r}k}$$

where [*A*] is the analyte mixing ratio, γ is the calibration factor, [*P* ^+^] is the product ion, [*R* ^+^] is the reagent ion, *t*_r_ is the reaction time, and *k* is the rate constant.

Calibration was performed for 12 VOCs and alkanes on the days sampling using multipoint standards covering the expected concentration range. Calibration curves were obtained via linear regression, and the resulting slopes were used as calibration factors to adjust analyte mixing ratios derived from eq 1. For species without standard gases, external calibration was not performed, and unadjusted mixing ratios from eq 1 were used. Further details on SIFT-MS calibration are provided in the Supporting Information.

Uncertainty for calibrated species was calculated by combining the standard error of the regression slope with uncertainties in the calibration equipment, including gas standard concentrations and the custom-built automated gas calibration unit (AGCU). The AGCU consists of (i) a heated VOC scrubber (palladium-coated alumina pellets heated to 380 °C) producing zero air while maintaining sample humidity, and (ii) mass flow controllers (Alicat) regulating flows of zero air and standard gas to achieve controlled dilution ratios from parts per trillion to parts per million (ppt-ppm). Automated stepwise dilution generated multipoint calibration curves for routine external calibration.

Due to the lack of available calibration standard gases, uncertainties could not be fully quantified for all measured VOCs. For these uncalibrated species, reported mixing ratios are subject to the systematic error (accuracy) inherent to SIFT-MS measurements, estimated at ±35%.^40,41^ This estimate reflects uncertainties associated with reaction rate, instrument calibration functions, and branching ratios, but does not include measurement noise (precision) or additional uncertainty arising from potential interferences (e.g., isomers and isobars). A complete total uncertainty is therefore not reported for noncalibrated species. Additional details on uncertainty calculations are provided in the Supporting Information Table S1 reports the average values of calibration factors for the full campaign, while Figure S1 shows their daily variability.

A zero offset was applied for all species, which was taken from zero air for VOCs and from a nitrogen blank. Additionally, we calculated the limit of detection (LOD) for all species from the signal at zero-level standard gases to ensure we measured a valid signal, not noise. The LODs for calibrated and noncalibrated species were calculated based on one standard deviation (1σ) and are provided in Table S1 along with their calibration factor and uncertainties.

Table 1 lists 11 masses (*m*/*z*) with their tentative compound formula assignments, along with potential interferences informed by previous studies cited in the reference column. These assignments consider potential interferences arising from fragmentation, isobaric or isotopic compounds, and expected VOC emission profiles. For instance, isobaric compounds (e.g., isoprene and furan at *m*/*z* 68, monoterpenes at *m*/*z* 136, and sesquiterpenes at *m*/*z* 204) may not be resolved by SIFT-MS and are therefore reported as the combined signal of all contributing compounds. Similarly, *m*/*z* 106 C_2_-alkylbenzenes represent the combined signal from all xylene isomers (ortho, meta, para) and ethylbenzene. Fragmentation can also produce interfering ions. For example, formaldehyde at *m*/*z* 31 (CH_3_O^+^) may include contributions from methanol and ethanol reactions with O_2_^+^.^42–44^

**Table 1:** Selected Species and Their Tentative Assignment^a^

*m*/*z*	reagent ion	product ion	tentative compound	tentative formula	potential interferences	ref
31	H_3_O^+^	CH_3_O^+^	formaldehyde	HCHO	methanol and ethanol	^43,46–49^
68	NO^+^	C_5_H_8_^+^	isoprene/furan	C_5_H_8_	*na*	^47,50^
78	NO^+^	C_6_H_6_^+^	benzene	C_6_H_6_	*na*	^47,51^
88	NO^+^	C_3_H_6_O·NO^+^	acetone	C_3_H_6_O	monoterpenes (25%), *n*-octane (5%)	^46,47,52^
102	NO^+^	C_4_H_8_O·NO^+^	butanone	C_4_H_8_O	*na*	^53^
106	NO^+^	C_8_H_10_^+^	C_2_–alkylbenzenes	C_8_H_10_	*na*	^47,51^
136	NO^+^	C_10_H_16_^+^	monoterpenes	C_10_H_16_	*na*	^1,47^
141	NO^+^	C_9_H_17_O^+^	nonanal	C_9_H_18_O	C_9_ ketones	^48,49,53,54^
142	NO^+^	C_7_H_12_O·NO^+^	2-heptenal	C_7_H_12_O	*na*	^49,55^
152	NO^+^	C_10_H_16_O^+^	citral	C_10_H_16_O	*na*	^1^
204	NO^+^	C_15_H_24_^+^	sesquiterpenes	C_15_H_24_	C_15_H_24_ petroleum hydrocarbon	^1,47,56^

a *m*/*z* is the ion molecule detected in SIFT-MS. *na* refers to unavailable or unknown interference.

SIFT-MS operates with relatively low energy, as excess energy is removed through collisions with nitrogen carrier gas, and no voltage gradient is applied along the flow tube. This results in less analyte fragmentation such that the protonation of formaldehyde is strongly favored over the deprotonation reaction. Moreover, the low kinetic energy of reactants in the SIFT-MS flow tube is the reason why the sensitivity of the instrument is not significantly impacted by relative humidity.^45^

While not all potential interferences are reported here, they are acknowledged as limitations of the method. The tentative compound assignments are intended to guide the interpretation of measured tracers and their likely emission sources rather than to provide the absolute values. Consequently, our discussion focuses on spatial patterns, tracers, and their correlations with indoor sources.

### Other Instruments

We also measured NO_*x*_ (NO + NO_2_) as the primary tracer of vehicle emissions. NO_*x*_ was measured using an Iterative Cavity-enhanced Differential Optical Absorption Spectrometer (ICAD, Airyx).^57^ ICAD directly measures NO_2_ in the spectral range between 430 to 465 nm, separating it from overlapping absorptions such as water vapor and glyoxal. The air sample first passes through an optical cavity for NO_2_ measurements. It then enters an ozone (O_3_) titration system, where NO is converted to NO_2_, before passing through a second optical cavity for total NO_*x*_ measurement. The spectrometer alternates between the two cavities every 2 s, providing NO_*x*_ mixing ratios at the same interval. Additional fast-response gas analyzers, including methane (CH_4_), carbon dioxide (CO_2_), O_3_, carbon monoxide (CO), and particulate matter (PM_2.5_ and PM_10_), were fitted in the WASP. These additional measurements, excluding NO_*x*_ are subject to discussion in a future study.

### Calculating Background and Increment Concentrations

To identify localized emissions, it is necessary to separate the background and local increment concentrations for each species. Background concentrations were estimated using the first percentile measurement within a rolling window from the mobile time series.^5,58^ In this study, a 5 min rolling window of 10 s observation data was applied, including data from 2.5 min before and after each data point. Using the first percentile ensures that even the smallest concentration increments are included in the analysis. The 5 min rolling window generates a robust baseline, minimizing the influence of localized emissions (Figure S2). Figure S3 shows the calculated background concentrations for *m*/*z* 88 (C_3_H_6_O·NO^+^; acetone), *m*/*z* 141 (C_9_H_17_O^+^; nonanal), and NO_*x*_ in Bradford and their total measured values. By subtracting the background from the total measured values, we obtain the incremental concentrations of these species, which would be expected to be more influenced by local sources.

The median concentration of each species from all drive circuits was calculated along the road network using a *distance-weighted median* approach, adapted from the *distance-weighted mean* method developed by Wilde et al.^5^ Median concentrations from repeat measurements were adopted because they are more likely to represent persistent sources, whereas mean values can be strongly affected by infrequent extreme events.^59^ In addition, because the geolocation of measured pollutants varied between campaigns and the route could slightly deviate during sampling, a set of uniformly spaced road points was generated based on the initial route design.

The *distance-weighted median* calculates the median concentration on measurement points spaced 30 m apart along the measurement driving circuit, applying weights calculated using the Gaussian kernel (eq 2)­

2 $$K\left(x,x^{\prime }\right)=\frac{1}{\sqrt{2\pi \sigma }}\exp\left(-\frac{{∥x-x^{\prime }∥}^{2}}{2\sigma^{2}}\right)$$

where *K*(*x*, *x*′) is the Gaussian kernel value, ∥*x* – *x*′∥^2^ is the squared Euclidean distance between the regression point (the 30 m interval road point) *x* and the observation point *x*′, and σ is the standard deviation that controls the width of the Gaussian curve. In this study, we used σ values of 50 and 100 m for the increment and background concentrations, respectively. The 30 m interval represents the spacing of road points along the measurement route used for plotting and applying the Gaussian smoother. This interval was chosen to capture the underlying geography of the road network and to make the computation faster compared to the smaller interval.

The Gaussian kernel assigns higher weights to measurements closer to a measurement point, with the weights decreasing as distance increases, reflecting the stronger influence of nearby emissions on the concentration at each network point. The Gaussian kernel smooths the data spatially to help reveal spatial differences in concentrations. Distances between network and measurement points were calculated using the Haversine (great-circle) distance. A smaller σ value narrows the Gaussian, capturing localized effects at a finer spatial scale, which helps distinguish the increment concentration from the background.

### Emission Source Factor

The potential contribution of different source types to the concentration of a species at any point depends on many factors, including source strength, source location, and the prevailing meteorological conditions. We consider three main types of emission sources: auto repair shops, beauty salons, and restaurants. These source types cover numerous individual premises, sizes, and levels of activity for which emissions information is unavailable. However, information is available on the locations of individual premises, which can be used to develop a *source factor*. Source locations were retrieved using the Google Maps API by searching source-based keywords. For restaurant emissions, keywords included “restaurant”, “cafe”, “fast food”, and “takeaway”. For beauty salon emissions, searches were based on terms like “beauty salon”, “beauty parlour”, and “nail salon”. For auto repair emissions, we used keywords such as “car repair”, “vehicle repair”, and “auto repair”. These searches resulted in 576 restaurants, 163 auto repair shops, and 256 beauty salons being identified across the Bradford city domain.

To develop a *source factor*, each individual source was modeled with a unit emission rate of 1 mg m^–3^ s^–1^ using the ADMS 5.0 model, an advanced Gaussian plume modeling system.^60^ Hourly meteorological data were obtained for the Bingley site, approximately 8.5 km northeast of Bradford center. As input, the ADMS model requires hourly sequential meteorological data consisting of wind speed, wind direction, ambient temperature, and cloud cover. Also required is the surface roughness length of both the meteorological measurement location (assumed to be 0.1 m) and that of the receptor locations in Bradford (assumed to be 0.5 m). These measured inputs are used by the ADMS meteorological preprocessor that is used to calculate variables for use in dispersion calculations, such as boundary layer height, friction velocity, and Monin-Obukhov length. Sources were represented as “shallow volumes” representative of emissions from a small building with dimensions 10 × 10 × 4 m (length × width × height). For each hour, all sources of a particular type, e.g., the 576 restaurants where modeled using ADMS to produce a concentration surface across the Bradford domain for each hour over the duration of the mobile measurements.

The outcome of these calculations provides a single *source factor* for any latitude-longitude for each source type and for each hour of the measurement campaign. The relationship between the *source factor* and the concentrations of different species can then be considered. The *source factor* is used to test the strength of the relationship between measured concentrations and the weighting factor. It is acknowledged that the *source factor* does not account for the magnitude of emissions from individual sources, which is unknown, but should provide a useful metric to relate measured concentrations to both the density and proximity of sources to the mobile measurement locations. For example, the *source factor* will be higher in areas where there is a high density of particular source types, such as restaurants in central Bradford. Further refinements could include accounting premises of different sizes, should such information become available.

### K-means Cluster and Generalized Additive Model Analyses

Potential sources of pollutants are often concentrated in commercial areas near the city center and along main roads. Spatial cluster analysis—an unsupervised classification technique for homogeneous characteristics—was employed to better understand the spatial distribution of sources based on their influential level (*source factor*). K-means clustering was applied to the entire data set of calculated *source factors* for beauty salons, restaurants, and auto repair shops to identify zones where these sources are the most influential. The analysis was performed using the *cluster* and *factoextra* R packages, and the four-cluster solution was determined from evaluating three methods, as described in SI and illustrated in Figure S5.

While clustering identifies groups with similar influence levels, it does not evaluate how these influences relate to observed pollutant concentrations. To address this, we applied a generalized additive model (GAM) approach^61^ to each cluster to evaluate the relationship between clustered *source factors* and incremental concentrations of indoor tracers. GAMs capture complex nonlinear relationships and demonstrate how increases in species concentrations correspond to increasing influence from specific sources. Further details on the GAM analysis are provided in the Supporting Information.

To ensure the robustness of the GAM-derived association and to account for measurement uncertainty, sample size, and data processing steps, we applied a bootstrap resampling approach (*n* = 100 iterations). For each source type and tracer, the data set was resampled with replacement, and a GAM was fitted to each bootstrap sample. We then calculated the median and the 2.5th and 97.5th percentiles of the resulting predictions across the range of *source factor* values to generate the 95% confidence intervals.

## Results and Discussion

### Spatial Distribution of VOCs

Repeated measurements along a dedicated mobile monitoring route revealed the spatiotemporal distribution of incremental concentrations of VOCs, as illustrated in Figure 2. The figure presents spatial and temporal patterns for *m*/*z* 88 (C_3_H_6_O·NO^+^; acetone), *m*/*z* 141 (C_9_H_17_O^+^; nonanal), and NO_*x*_—three out of 35 species measured in Bradford. The color gradients indicate concentration variation across measurement circuits, apparent in individual afternoon, evening, and morning measurements selected from the 22 measurement circuits, which show 10 s measurements. The left panel of Figure 2 shows concentration at 30 m interval points calculated using the *distance-weighted median* approach. These *distance-weighted median* plots were useful for identifying locations where each VOC showed elevated concentrations, which then informed the identification of potential local sources for estimating *source factors*. While we report estimated mixing ratios in ppb, our primary focus is on identifying spatial patterns of the measured species and their associations with indoor activity sources.

**Figure 2:**
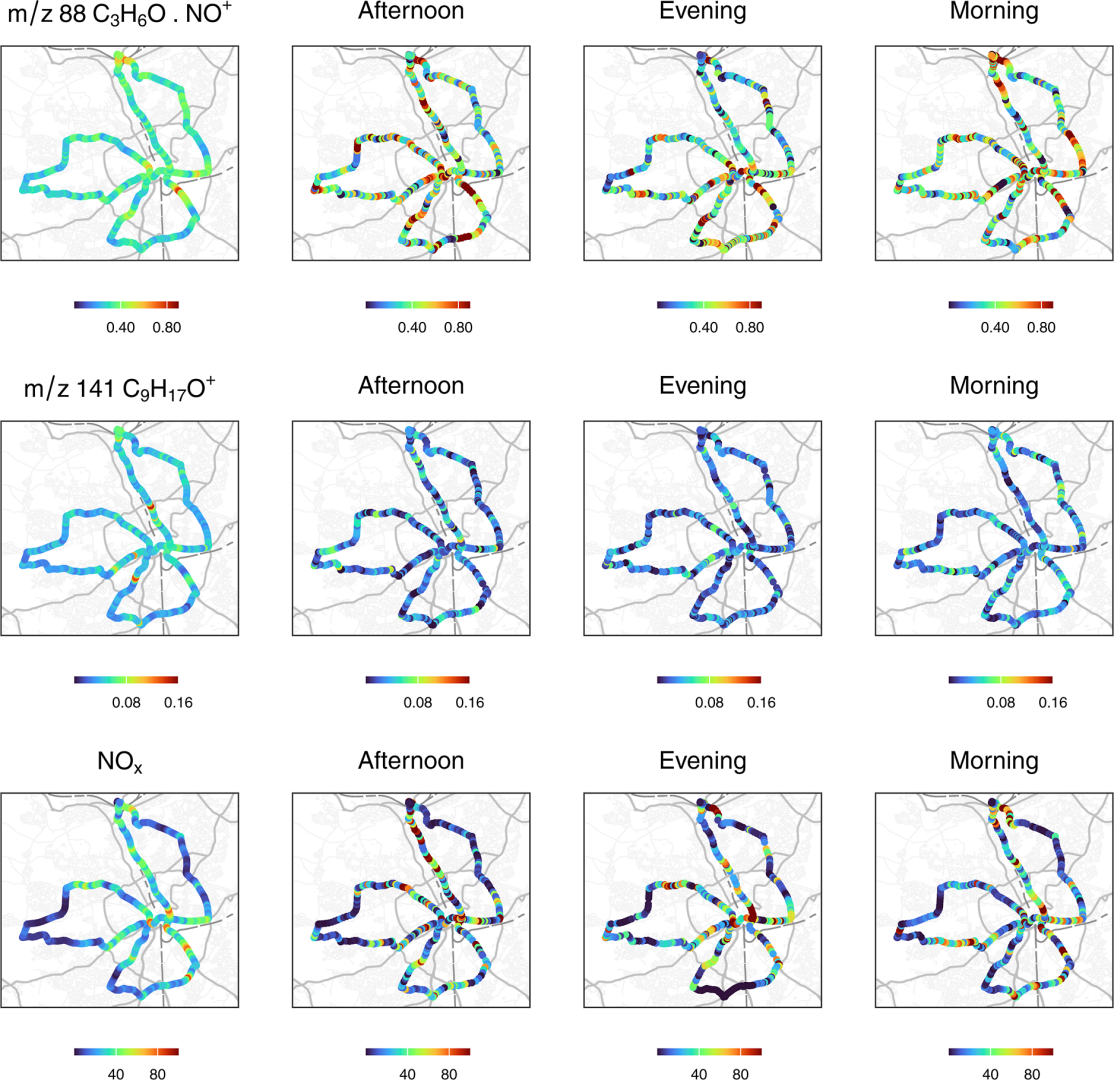
Spatial distribution of (left-most panel) the *distance-weighted median* spatial concentration and concentrations measured during three individual drives: afternoon (28/02/2023, 13:00–15:00), evening (28/02/2023, 17:00–19:00), and morning (01/03/2023, 10:00–12:00). Shown are incremental concentrations in parts per billion (ppb) for *m*/*z* 88 (C_3_H_6_O·NO^+^; acetone), *m*/*z* 141 (C_9_H_17_O^+^; nonanal), and NO_*x*_. Median concentrations are plotted at 30 m observation point intervals created on the predefined driving circuit. Individual-drive panels show 10 s incremental concentrations.

Acetone and nonanal exhibit higher concentrations in some locations, whereas elevated NO_*x*_ levels are more prevalent on major roads and intersections. By repeating measurements along the same route at different times and on different days—with minimal route deviation—we were able to identify persistent emission trends beyond what single-pass measurements would reveal, as suggested by Kerckhoffs et al.^62^ The use of a dedicated route also allowed us to distinguish background levels from persistent concentration increments.

The *distance-weighted median* method provides insights into the spatial distribution of weighted-median incremental concentrations, as shown in Figure 2. This approach highlights locations where specific pollutants consistently exhibit high incremental concentrations, independent of time or season, while minimizing the effect of isolated or infrequent events. Differences in the spatial distributions of incremental concentrations for the three species likely reflect variation in their source influences.

While weighted-median incremental concentrations help identify persistent pollutant hotspots, they do not establish a direct link between indoor sources and observed pollutants in the ambient atmosphere. Indoor sources often consist of complex mixtures that, upon emission to the ambient atmosphere, disperse and mix with outdoor emissions, such as vehicle exhaust in urban environments. For example, compounds like benzene, toluene, and xylene can originate from vehicle emissions,^34^ cooking exhausts,^36^ and auto repair shops,^35^ making source identification challenging.

Furthermore, intercorrelating species across all measurements proves ineffective due to overlapping emission profiles among source types. For instance, benzene, toluene, and xylene may show strong interspecies correlations in traffic-congested areas, but similar correlations can also appear in areas with less traffic but nearby restaurants or auto repair shops. This underlines the need for a spatially aggregated source apportionment approach to better understand the contribution of indoor emissions to ambient pollution.

### Spatially Aggregated Sources Contribution

Spatially aggregated source characterization was conducted by calculating *source factors* across the study area for each main source type. This calculation extended beyond the mobile measurement route to cover Bradford City and its surrounding towns and villages. The resulting maps in Figure 3 show the contributions of three source types—auto repair shops, beauty salons, and restaurants—as mean source-specific values for each latitude-longitude coordinate averaged across all hours of the measurement campaigns.

**Figure 3:**
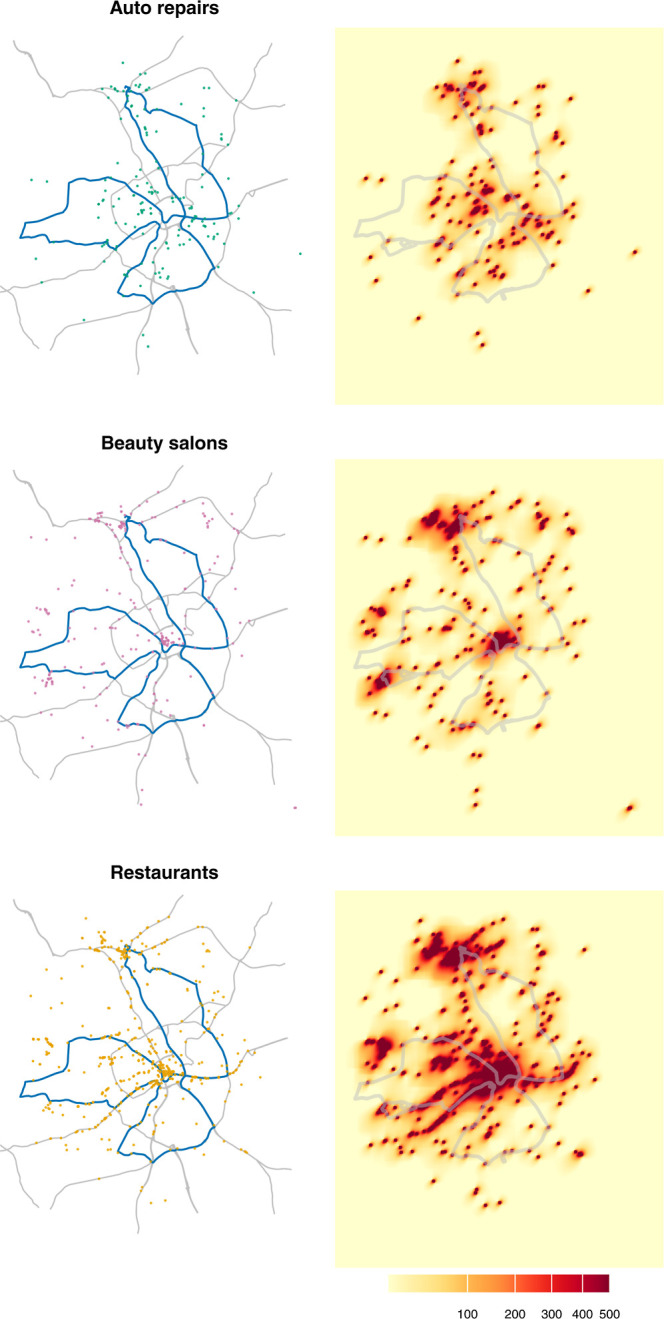
Spatial distribution of commercial sources in study area, Bradford, England. Left panels: Discrete point-source locations for auto repair shops (*n* = 163), beauty salons (*n* = 256), and restaurants (*n* = 576) used as ADMS model input. Right panels: Modeled *source factor* intensity at 10 m resolution grid averaged across all measurements (57 h). The heatmaps use a normalized scale to illustrate the regional extent of plume dispersion.

As shown in Figure 3, the spatial distribution of *source factors* reflects the high-density of commercial activity in Bradford city center and along major arterial roads. Restaurant hotspots are concentrated in Bradford city center (the center of the figure) and nearby town centers to the north–northwest. Beauty salon hotspots largely overlap with restaurant locations, with an additional hotspot in western Bradford (approximately −1.82° and 53.78°). In contrast, auto repair shop hotspots are more dispersed and less concentrated than those of restaurants and beauty salons.

While hotspots for all three source types often overlap due to urban land use patterns, particularly in the city center and north–northwest corridors, the relative intensity and spatial distribution of these factors differ based on the specific source locations (shown in the left panels of Figure 3). To visualize the broad spatial extent of these *source factors*, a normalized color scale is used. While this highlights the general areas influenced by emissions, it may visually reduce the order-of-magnitude differences in absolute source density between restaurants (*n* = 576) and auto repair shops (*n* = 163). Nevertheless, these local density gradients provide sufficient spatial variation for the GAM analysis to distinguish unique source-specific tracers from the aggregate urban background.

### Exploring Associations between Incremental Concentrations and *Source Factors*

By clustering the three *source factors*, we can identify areas that are most similar or dissimilar to one another based on their influence level. This approach is useful for identifying regions influenced by one source type while minimizing influence from others—for example, areas with high density of restaurants but with minimal contributions from beauty salons and auto repair shops.

The spatial clustering of the three source types was analyzed using K-means clustering across 22 measurement circuits (details in the Supporting Information). K-means is a well-established method that iteratively assigns each data point to the nearest cluster center and then updates the center based on the mean of the assigned points^63^ and is widely used to identify air pollutant sources, mostly at fixed monitoring or sampling stations.^64^ For example, Saksena et al.^65^ used a clustering method on air quality monitoring stations for SO_2_, NO_2_, and suspended particulate matter. However, their approach could not directly link pollutants to sources due to the complex mixtures and overlapping emissions from different sources. As shown in Figure S5C, clusters 1, 2, and 3 are primarily associated with auto repair shops, restaurants, and beauty salons, respectively. Cluster 4 is a mixed-source cluster associated with tracers from auto repair shops and restaurants. The cluster analysis (Figure S6B) reveals that auto repair shops, restaurants, and mixed-source clusters exhibit considerable overlap, while the beauty salon cluster is distinct. This outcome is expected owing to similarities in emission profiles between auto repair shops and restaurants, which are discussed in the following sections.

To quantify the relationship between these spatial influences of *source factors* and pollutant incremental concentrations, GAMs were applied to each cluster. The purpose of this step was to establish the extent to which different species concentrations increased with *source factor* values and whether the increases tended to be linear in nature. GAMs were selected over linear regression for their ability to capture complex relationships between predictors (*source factor*) and responses (tracer concentration). Tracers exhibiting positive relationships, where incremental concentrations increase with the *source factor* intensity, are presented in Figure 4.

**Figure 4:**
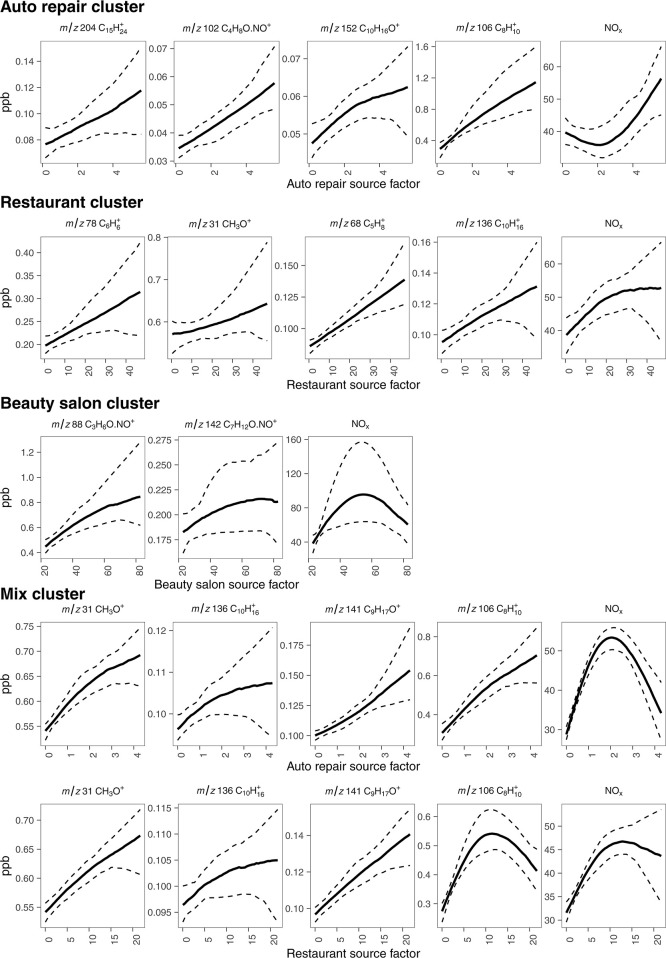
Relationships between tracer concentrations and *source factors* characterized using GAM fits. Panels are grouped by clusters influenced by (top to bottom): auto repair shops, restaurants, beauty salons, and mixed-source emissions. Solid lines represent the GAM-fitted median, and dashed lines represent the 95% confidence intervals derived from bootstrap resampling (*n* = 100 iterations). The total number of observations for each fit ranges from *N* = 1848 to *N* = 43,355. The bootstrap approach confirms the stability of the trends against individual extreme values and measurement and sampling uncertainties. See Supporting Information (Figures S6–S9) for corresponding fits of *source factors* that do not show positive correlations.

To address and quantify the uncertainties in the GAM relationships, comprehensive bootstrap simulations have been conducted. These uncertainties have been derived by randomly sampling the original data (with replacement) through fitting a GAM model. This process was repeated 100 times, resulting in 100 GAM predictions for each source factor and species combination. These 100 predictions were used to calculate the mean and 95% confidence interval of the relationship between the species concentration and *source factor*. This resampling approach has the benefit of implicitly reflecting uncertainties related to measurement and sampling uncertainties and the steps involved in deriving the GAM relationships. The bootstrap simulations yielded smooth trends and confidence intervals, shown in Figure 4, which highlight the strength or otherwise of the relationships between *source factors* and species concentrations. Table S2 associates the identified tracers with published emission fingerprints. Additional sensitivity analyses (Figures S6–S9) illustrate cases with weaker or absent relationships, ensuring a transparent characterization of the model’s predictive limits.

#### Auto Repair Cluster

Spatially, auto repair shops are dispersed across Bradford rather than being concentrated in the city center (Figure 3). Their proximity to central areas nonetheless contributes to elevated concentrations of the aforementioned compounds in adjacent neighborhoods, consistent with the spatial pattern observed for Cluster 1 (Figure S5C).

While compounds, including *m*/*z* 102 (C_4_H_8_O·NO^+^; butanone) and *m*/*z* 106 (C_8_H_10_^+^; C_2_-alkylbenzenes) show statistically significant enhancements, species such as *m*/*z* 152 (C_10_H_16_O^+^; citral) and *m*/*z* 204 (C_15_H_24_^+^; sesquiterpenes) exhibit more tentative positive associations with the *auto repair source factor* (Figure 4). For these species, the 95% confidence intervals overlap with the baseline at high *source factor* levels, likely due to low sampling density at the extremes of the influence range. However, their presence is consistently mapped to the auto repair cluster and aligns with known car-care product profiles (Table S2), suggesting they remain useful, albeit exploratory, markers for this source. Additionally, NO_*x*_ shows no discernible association with this *source factor*, indicating that the auto repair shop cluster variability is not driven by traffic-related emissions.

Figure S6 further demonstrates a lack of associations between these species and the *restaurant* and *beauty salon source factors*, confirming the interpretation that activities in auto repair shops are the dominant contributors to their concentrations within this cluster. The positive relationships observed for butanone and C_2_-alkylbenzenes are consistent with the use of solvents and fuels commonly associated with vehicle exhaust, repair, and air freshener.^51,66–68^ Additional associations with citral and sesquiterpenes likely reflect the use of fragranced products, including essential oils used in car disinfectant sprays^69,70^ and surface cleaning agents,^71^ within auto repair shops.

#### Restaurant Cluster

Spatially, this cluster is adjacent to the Auto Repair Cluster (Figure S5C) and is characterized by a high density of takeaways, cafes, and restaurants along major streets and roads in Bradford (Figure 3). This colocation with traffic corridors likely contributes to the weak association and greater uncertainty observed with NO_*x*_, reflecting mixed influences from cooking-related emissions^72^ and other combustion sources (e.g., residential gas heating),^73^ rather than direct attribution to the traffic source alone.

Species, including *m*/*z* 68 (C_5_H_8_^+^; isoprene/furan), exhibit a statistically significant enhancement, while *m*/*z* 31 (CH_3_O^+^; formaldehyde), *m*/*z* 78 (C_6_H_6_^+^; benzene), and *m*/*z* 136 (C_10_H_16_^+^; monoterpenes) show more exploratory associations with the *restaurant source factor* (Figure 4). While benzene and monoterpenes are known constituents of cooking emissions,^1,47,48^ benzene is ubiquitous in the urban background (e.g., traffic, combustion),^51^ making it difficult for the model to isolate it as a unique restaurant tracer (Table S2). Similarly, formaldehyde has been detected in car freshener^68^ and byproducts of combustion and high-heat cooking.^1,48^ Signal at *m*/*z* 68 likely reflects a combination of isoprene (associated with human breath and food preparation^1,74^ ) and furan (a product of heated cooking oils^75^), which cannot be distinguished at this nominal mass.

In contrast, these compounds exhibit weak or absent relationships with the *auto repair* and *beauty salon source factors* (Figure S7), indicating that they do not covary with those sources. This difference further supports the interpretation that restaurant-related activities dominate the observed tracers in this cluster, despite partial spatial overlap with or other source types.

#### Beauty Salon Cluster

The beauty salon cluster is concentrated in the center of Bradford and the adjacent town of Shipley (Figures 3 and S5C). While acetone shows a statistically significant enhancement, 2-heptenal exhibits a more tentative association with the *beauty salon source factor*. Both tracers show substantially weaker correlations with the restaurants and auto repair factors (Figure S8).

Acetone is a common ingredient of nail and cosmetic products and is widely reported in beauty salons indoor air.^16,76,77^ Similarly, 2-heptenal is an oxidation product of botanical extract (e.g., walnut oil) commonly used in skincare product formulations,^78,79^ supporting its attribution to salon activities. Despite the central urban location of many salons 1, NO_*x*_ exhibits a weak and highly uncertain relationship with this factor, suggesting that the identified tracers are linked to salon-specific activities rather than general traffic.

The lower number of identified tracers in this cluster likely reflects limited sampling coverage immediately surrounding salon locations compared to the more ubiquitous restaurant sites.

#### Mixed-Source Cluster

As shown in Figure S5C, the mixed-source cluster is the largest identified spatial region, suggesting overlapping influences from multiple urban emission sources. Several compounds, including formaldehyde and *m*/*z* 141 (C_9_H_17_O^+^; nonanal), exhibit statistically significant enhancements associated with both the *auto repair* and *restaurant source factors*. In contrast, C_2_-alkylbenzenes show significant enhancement only with the *auto repair source factor*. Monoterpenes do not show significant enhancement with either factor in this cluster, despite their tentative association observed in the dedicated restaurant cluster.

These results reflect a blending of the chemical signatures identified in earlier sections: while C_2_-alkylbenzenes remain a robust marker for auto repair activities, formaldehyde and monoterpenes appear as shared tracers across both source types. Nonanal, in particular, originates from the oxidation of oleic acid in cooking oils,^1,80^ but it is also emitted from vehicle interior materials.^81,82^ Its positive relationships with both auto repair and restaurant factors provide a physical basis for its classification within the mixed-source cluster.

Future work could further refine the interpretation of the relationships between *source factors* shown in Figure 4. While the main purpose of these models is to examine how individual VOC concentrations relate to their respective *source factors*, future work exploring VOC ratios from these relationships could further elucidate specific source contributions. Furthermore, other urban activities not explicitly resolved here—such as vaping—may contribute to compounds like monoterpenes and represent a unique source of propylene glycol.^83^ The absence of propylene glycol measurements in this study precludes explicit identification of such sources, highlighting a key limitation and opportunity for future study.

## Strengths, Limitations, and Future Directions

This study introduces a novel *source factor* approach that combines an extensive range of chemical species measurements with repeated mobile observations. A key strength of our study lies in the use of a designated monitoring route and extensive repeat measurements, which distinguish incremental concentrations from background levels. A total of 35 species were monitored across 22 measurement circuits (approximately 56 ADMS-modeled hours) under varying meteorological conditions, days of the week, and times of day, with 11 of these species further linked to their potential indoor emission sources. This combination of species diversity, temporal coverage, and spatial resolution is unique, allowing us to identify localized and persistent VOC emissions and link them to specific indoor source types.

Unlike most studies that focus on indoor VOC concentrations within the premises, this work evaluates emissions as detected outdoors through mobile monitoring. Since atmospheric processing and dispersion can significantly reduce VOC concentrations after release, linking observed tracers back to their sources becomes challenging. For instance, while the average concentration of acetone inside nail salons has been reported at 6 ppm,^84^ our measurements in the beauty salon cluster captured outdoor incremental concentrations up to 1 ppb (Figure 4), consistent with previous observations in London.^85^ Owing to the rapid chemical composition analysis conducted in this study, low-level tracer emissions were detected prior to extensive atmospheric transformation or dispersion. Furthermore, repeated measurements under varying conditions allowed for the identification of persistent VOC signatures specific to certain source types.

This approach also facilitates data filtering to isolate locations predominantly influenced by specific source types, complementing receptor modeling for interpreting measurement data and the relationships between tracers and their sources. For example, within the beauty salon cluster, acetone was strongly correlated with the respective *source factor* (Figure 4), but not with the *restaurant* or *auto repair* *source factors* (Figure S8).

The limitation of the *source factor* approach is its inability to account for the emission magnitude from individual sources—for example, emissions from takeaway versus dine-in restaurants. Nevertheless, the combination of rapid and repeated measurements enabled the detection of sources with relatively low tracer emissions. Despite this simplified approach, the consistent correlations observed between incremental tracer concentrations and their respective *source factors* suggest that the method is effective in capturing key source-tracer relationships along the measurement route.

In addition, the *source factor* metric does not explicitly account for heterogeneity within each source category and temporal variations in their emission signatures. For instance, differences in cooking fuel types and styles of the restaurant may introduce variability in the emitted VOC profiles. Future work could address this by incorporating on-site source sampling for source chemical fingerprint characterization.

Future targeted source studies can build on the *source factor* metric developed here. The *source factor* heatmap (Figure 3) could be further refined by broadening the range of indoor source types and accounting for premise size where information is available, and extending the scope of geographic database queries. This would help optimize and prioritize measurement routes to maximize particular source contributions. Additionally, the *source factor* heatmap can inform sampling strategies (on-site in addition to mobile) and instrument selection to better capture source-specific tracers—for example, using Proton Transfer Reaction-Mass Spectrometry (PTR-MS) to improve the detection of siloxanes.^9^

Further, investigating the spatial decay of tracer concentrations from high-density source locations could provide further insights into temporal emission characteristics. For restaurant emissions, in particular, extending measurement periods later into the evening would capture varying activity hours, as revealed in the Las Vegas, USA study.^9^

Finally, this methodology is adaptable to other source types, provided their geographical locations are known, making it a valuable tool for assessing existing emissions sources and predicting the impact of future sources on local air quality.

## Supplementary Material



## Notes

The Supporting Information includes: details of data collection (Table S1) and calibration procedures (Figure S1); background concentrations of selected species at different rolling windows (Figure S2); spatial distributions of selected species over 22 measurement circuits (Figure S3); methods used to determine the optimal number of clusters (Figure S4); summary of four-cluster analyses (Figure S5); and GAM results showing correlations between tracer species and *source factors* that are not highly influential for each cluster (Figures S6–S9) (PDF)
