# Supplementary material for: Toward Linking Indoor Commercial Source Emissions to Outdoor Volatile Organic Compounds Using Mobile Measurements
Source: ACS EST Air. 2026 Apr 22;3(5):1191–203. doi: 10.1021/acsestair.5c00290 (PMC13162265; doi:10.1021/acsestair.5c00290)
Supplement: Supplementary file 1 [file ea5c00290_si_001.pdf]

Supporting Information: Toward Linking Indoor Commercial Source Emissions to Outdoor Volatile Organic Compounds Using Mobile Measurements

Sri Hapsari Budisulistiorini,* Thomas C. Moore, Marvin D. Shaw, Will S. Drysdale, James D. Lee, and David C. Carslaw*

Wolfson Atmospheric Chemistry Laboratories, Department of Chemistry, University of York, York, YO10 5DD, United Kingdom

E-mail: sari.budisulistiorini@york.ac.uk; david.carslaw@york.ac.uk

Summary

Number of pages: 20

Number of Figures: 9

Number of Tables: 2

Supporting Information Available

The following files are available free of charge.

Figures

Figure S1: The calibration factor (the slopes of calibration curves) from the standard VOC and alkanes from SIFT-MS calibrations in winter and summer 2023.

Figure S2: Time series of acetone background concentration from one driving loop in Bradford, calculated as the 1st percentile in rolling 2.5, 5, and 10 min windows. The measured concentration is overlaid in gray color.

Figure S3: Spatial distribution of acetone, nonanal, and NO_x, representative tracers of personal care products, cooking, and traffic emissions. The top and bottom panels depict the background and measured concentration of the tracers from individual driving circuits, respectively. Background concentrations were calculated using the *rolling background* method. Larger marker sizes indicate values exceeding the scale.

Figure S4: Three methods for determining the optimal K-Mean Clusters calculated for three types of emission sources. The dashed line indicates the suggested optimal number of clusters for each method. The orange dashed line in (A) represents the selected number of clusters. These clusters were used to analyze the clusters of sources and their relationship with the increment concentration of VOCs.

Figure S5: (A) Number of variables in each cluster. Each variable represents observation points from all measurement circuits, characterized by the sum of the weighting factor indicating source contribution. (B) Principal component analysis plot showing variables from each cluster, based on the first two principal components that capture the majority of the variance. (C) Spatial distribution of clusters mapped onto their corresponding locations along the measurement circuit.

Figure S6: Additional GAM fits between *m/z* 102 ($C_4H_8O \cdot NO^+$; butanone), *m/z* 106

($C_8H_{10}{}^+$; C_2-alkylbenzenes), *m/z* 152 ($C_{10}H_{16}O^+$; citral), and *m/z* 204 ($C_{15}H_{24}{}^+$; sesquiterpenes) and (A) beauty salon and (B) restaurant *source factors*. The lack of positive correlation indicates that these species do not co-vary with either *source factors*, suggesting minimal contribution from beauty salons or restaurants.

Figure S7: Additional GAM fits between *m/z* 31 (CH_3O^+; formaldehyde), *m/z* 68 ($C_5H_8{}^+$; isoprene/furan), *m/z* 78 ($C_6H_6{}^+$; benzene), and *m/z* 136 ($C_{10}H_{16}{}^+$; monoterpenes) and (A) auto repair and (B) beauty salon shop *source factors*. The lack or absence of positive correlation and larger uncertainty indicate that these species do not co-vary with either *source factors*, suggesting minimal contribution from auto repair shops or beauty salons.

Figure S8: Additional GAM fits between *m/z* 99 ($C_3H_6O \cdot NO^+$; acetone) and *m/z* 142 ($C_7H_{12}O \cdot NO^+$; 2-heptenal) and (A) *auto repair* and (B) *restaurant source factors*. The absence of positive correlation and larger uncertainty indicates that these species do not co-vary with either *source factors*, suggesting minimal contribution from auto repair shops or restaurants.

Figure S9: Additional GAM fits between *m/z* 31 (CH_3O^+; formaldehyde), *m/z* 136 ($C_{10}H_{16}{}^+$; monoterpenes), *m/z* 141 ($C_9H_{17}O^+$; nonanal) and *m/z* 106 ($C_8H_{10}{}^+$; C_2-alkylbenzenes) and the *beauty salon source factor*. The absence of correlations indicates no direct contribution from beauty salon activities to their concentration.

Tables

Table S1: Masses measured by SIFT-MS along with their reagent and product ions, and means of the limit of detection (LOD), calibration factor, and uncertainties.

Table S2: Summary of source-tracer associations with published emission profiles.

SIFT-MS Calibration and Uncertainties

Calibration was performed for 12 species by introducing known concentrations of standard gas into the SIFT-MS and associating the delivered concentration with the measured response using linear regression:

$$C_{meas} = \beta_1 C_{del} + \beta_0 \tag{1}$$

where C_{meas} is the measured response, C_{del} is the delivered standard gas concentration, β_1 is the slope of the regression line (the calibration coefficient), and β_0 is the intercept, assumed to be zero (i.e., no signal in the absence of a calibrant).

The total relative uncertainty (u_{rel}) for each calibrated species was calculated by combining the standard error of the slope (SE_{β_1}) with uncertainties from calibration equipment, namely the standard gas concentration (u_{gas}) and gas blender precision (u_{blend}):

$$u_{rel} = \sqrt{\left(\frac{SE}{\beta_1}\right)^2 + u_{gas}^2 + u_{blend}^2} \tag{2}$$

The absolute uncertainty for each species (u_{species}, in ppb) was then obtained from: $u_{species} = C_{meas} u_{rel}$. Here, u_{gas} varied between 5-20%, while u_{blend} was approximately ±2%. Table S1 summarizes the calibration results, including β_1 (slope), SE_{β_1} (slope SE), u_{rel} (uncertainty in %), and u_{species} (uncertainty in ppb).

Not all species could be calibrated due to the absence of the standard gases. The inherent instrument error representing the accuracy is reported to be ± 35% was assumed.[1,2] With the lack of standard gases, we could not measure the measurement precision, and thus could not quantify the uncertainty of the non-calibrated species.

Spatial K-means cluster analysis

Spatial K-means cluster analysis was applied to the entire dataset to better understand the relationship between *source factors* and the incremental concentrations of indoor tracers. This analysis produced groups of sources with similar potential contributions to outdoor emissions, as indicated by their *source factor* values.

For m data $x_1, x_2, \ldots, x_m \in R^n$, K-means clustering algorithm proceeds as follows:[3,4]

1. Choose the number of clusters. Specify the number of cluster (l, where l ¡ m (in this study $l = 10$).

2. Initialize cluster centroids. Randomly select l points from the feature space as the initial centroids $c_1, c_2, \ldots, c_l$. Compute the distances between each centroid and all data points.

3. Assign points to the nearest cluster. Each data point x_i is assigned to the cluster with the nearest centroid, according to the minimum distance criterion:

$$x_i \in C_{j^*} \quad \text{if} \quad \|x_i - c_{j^*}\| = \min_{1 \leq j \leq l} \|x_i - c_j\|$$

4. Update centroids. For each cluster C_j, update the centroid as the mean of the points assigned to that cluster:

$$c_j = \frac{1}{m_j} \sum_{x_i \in C_j} x_i, \quad 1 \leq j \leq l,$$

   where m_j is the number of points in cluster C_j.

5. Iterate until convergence. Repeat steps 3-4 until cluster assignments no longer change or the change in centroids falls below a predefined threshold.

The optimal number of clusters was determined by evaluating these methods.[3,5,6]

1. Elbow method. This method (Figure S4-A) evaluates the total within-cluster sum of squared error (SSE), defined as the sum of squared distances between each point and its cluster centroid. As the number of clusters increases, SSE decreases, but the rate of improvement drops after a certain point. The "elbow" corresponds to the number of clusters beyond which additional clusters yield only marginal gains, indicating an appropriate choice for k.

2. Silhouette method. This method (Figure S4-B) measures how similar each point is to its own cluster compared to other clusters. The silhouette coefficient ranges from -1 to $+1$, with values close to $+1$ indicating well-separated clusters, values near 0 suggesting overlapping clusters, and negative values indicating possible misclassifications. Average silhouette values above 0.5 suggest a reasonable structure, while values below 0.25 imply weak or no meaningful clustering.

3. Gap statistic method. This method (Figure S4-C) compares the within-cluster dispersion of the observed data to that expected under a null reference distribution (i.e., data with no obvious clustering structure). The optimal number of clusters is chosen as the smallest k for which the observed dispersion is significantly smaller than that expected under the null model. A larger gap statistic indicates better clustering, meaning the data form clearer groups than would be expected by chance.

Based on the combined evidence from the three methods, four clusters were selected according to the Elbow method. Although this differs from the Silhouette method (two clusters) and Gap Statistic method (ten clusters), the four-cluster solution provided the most meaningful interpretation of the observed sources. Specifically, it captures three distinct indoor sources and one potentially mixed-source cluster in Bradford. The mixed-source cluster overlaps with two others, which likely reduced its resolution in the Gap Statistic method, but retaining it as a separate cluster better reflects the source contributions. Therefore, despite minor discrepancies with other statistical criteria, the four-cluster solution offers the most

interpretable characterization of the source cluster in Bradford.

Generalized additive model

A generalized additive modeling (GAM) approach was employed to describe the relationship between the source influences, represented by the *source factor*, and the incremental concentrations of indoor air tracers measured outdoors. This approach was chosen because it provides greater flexibility than linear models, which may be too restrictive when the relationships between explanatory variables (e.g., emission sources) and pollutant concentrations are complex and nonlinear.

The additive model is expressed:[7,8]

$$C_i = \sum_{j=1}^{n} s_j(x_{ij}) + \varepsilon_i, \tag{3}$$

where C_i is the ith concentration of the time series, $s_j(x_j)$ is a smooth function of the jth covariate, n is the total number of covariates, and ε_i is the residual error term, assumed to follow a normal distribution with variance σ^2.

The analysis was conducted in *R*, by specifying the ”gam” method in *ggplot2::stat_smooth* function. A basis dimension of 3 was applied to the smooth term to provide sufficient flexibility while avoiding overfitting.

Table S1: Masses measured by SIFT-MS along with their reagent and product ions, and means of the limit of detection (LOD), calibration factor, and uncertainties.

m/z	**Reagent ion**	**Product ion**	**LOD**	**External cal.**	**Slope**	**Slope SE**	**Uncert. (%)**	**Uncert. (ppb)**
28	O_2^+	$C_2H_4^+$	0.19	yes	13.87	0.29	20	4.49
31	H_3O^+	CH_3O^+	0.57	no	*na*	*na*	*na*	*na*
33	H_3O^+	CH_5O^+	0.77	yes	1.04	0.17	26	7.22
35	H_3O^+	H_3S^+	0.72	no	*na*	*na*	*na*	*na*
42	H_3O^+	$CH_3CN \cdot H^+$	0.22	yes	0.99	0.04	21	4.4
45	H_3O^+	$C_2H_4O \cdot H^+$	0.31	no	*na*	*na*	*na*	*na*
47	H_3O^+	$C_2H_7O^+$	0.41	yes	1.29	0.1	22	2.76
49	H_3O^+	$CH_4S \cdot H^+$	0.09	no	*na*	*na*	*na*	*na*
54	NO^+	$C_4H_6^+$	0.03	no	*na*	*na*	*na*	*na*
55	NO^+	$C_3H_3O^+$	0.37	no	*na*	*na*	*na*	*na*
58	O_2^+	$C_4H_{10}^+$	0.88	yes	2.16	0.03	20	32.73
68	NO^+	$C_5H_8^+$	0.08	yes	0.42	0	5	0.86
69	NO^+	$C_4H_5O^+$	0.03	no	*na*	*na*	*na*	*na*
78	NO^+	$C_6H_6^+$	0.11	yes	0.61	0.01	12	3.01
88	NO^+	$C_3H_6O \cdot NO^+$	0.55	yes	0.47	0.04	23	5.14
88	O_2^+	$C_4H_8O_2^+$	1.06	no	*na*	*na*	*na*	*na*
92	NO^+	$C_7H_8^+$	0.08	yes	0.52	0.01	7	1.68
102	NO^+	$C_4H_8O \cdot NO^+$	0.1	no	*na*	*na*	*na*	*na*
106	NO^+	$C_8H_{10}^+$	0.1	yes	0.63	0.01	6	1.25
120	NO^+	$C_9H_{12}^+$	0.13	yes	0.52	0.01	9	1.41
126	NO^+	$C_5H_4O_2 \cdot NO^+$	0.05	no	*na*	*na*	*na*	*na*
129	NO^+	$C_8H_{17}O^+$	0.04	no	*na*	*na*	*na*	*na*
136	NO^+	$C_{10}H_{16}^+$	0.16	yes	0.3	0.01	20	2.32
139	NO^+	$C_{10}H_{19}^+$	0.07	no	*na*	*na*	*na*	*na*
141	NO^+	$C_9H_{17}O^+$	0.04	no	*na*	*na*	*na*	*na*
142	NO^+	$C_7H_{12}O \cdot NO^+$	0.13	no	*na*	*na*	*na*	*na*
150	NO^+	$C_9H_{10}O_2^+$	0.09	no	*na*	*na*	*na*	*na*
152	NO^+	$C_{10}H_{16}O^+$	0.07	no	*na*	*na*	*na*	*na*
160	NO^+	$C_7H_{14}O_2 \cdot NO^+$	0.08	no	*na*	*na*	*na*	*na*
176	NO^+	$C_{13}H_{20}^+$	0.04	no	*na*	*na*	*na*	*na*
204	NO^+	$C_{15}H_{24}^+$	0.18	no	*na*	*na*	*na*	*na*
225	NO^+	$(Si{-}283\,O_3(CH_{35}(OH_2^+$	0.5	no	*na*	*na*	*na*	*na*
239	NO^+	$(Si{-}283\,O_2(CH_{37}(OH_2^+$	0.11	no	*na*	*na*	*na*	*na*
299	NO^+	$C_7H_{21}O_4(Si{-}284.\,H_2O^+$	3.27	no	*na*	*na*	*na*	*na*
355	NO^+	$C_9H_{27}O_5(Si{-}285\,^+$	1	no	*na*	*na*	*na*	*na*

m/z is the ion molecule detected in SIFT-MS. LOD is the limit of detection. Slope SE is the standard error of slope.
na refers to unknown values due to unavailable gas standards, resulting in unknown calibration factors and uncertainties.
The inherent systematic error (accuracy) of SIFT-MS is approximately ± 35%.[1,2]

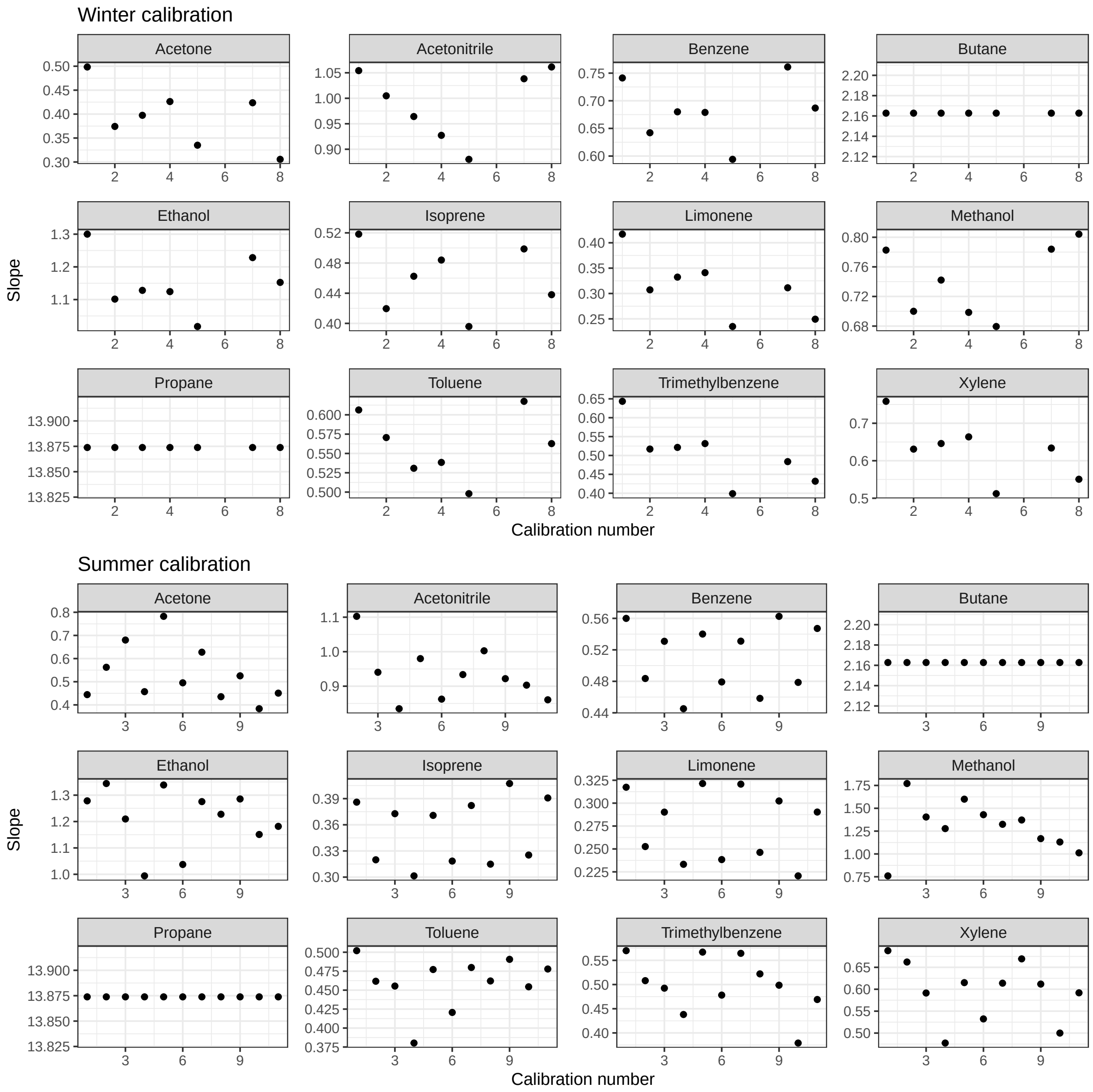

Figure S1: The calibration factor or the slopes of calibration curves from the standard VOC and alkanes from SIFT-MS calibrations in winter and summer 2023.

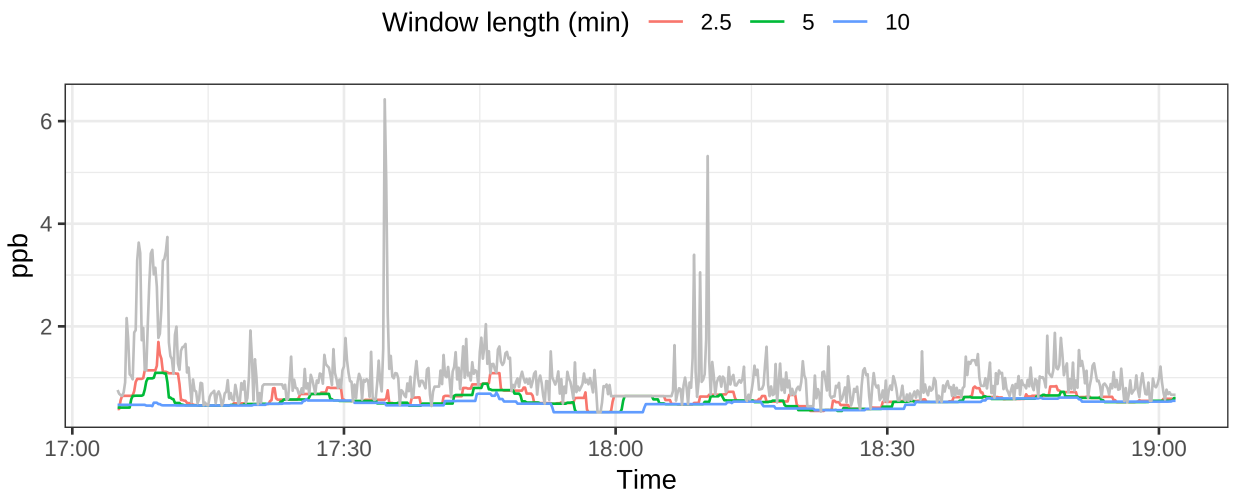

Figure S2: Time series of acetone background concentration from one driving loop in Bradford, calculated as the 1st percentile in rolling 2.5, 5, and 10 min windows. The measured concentration is overlaid in grey color.

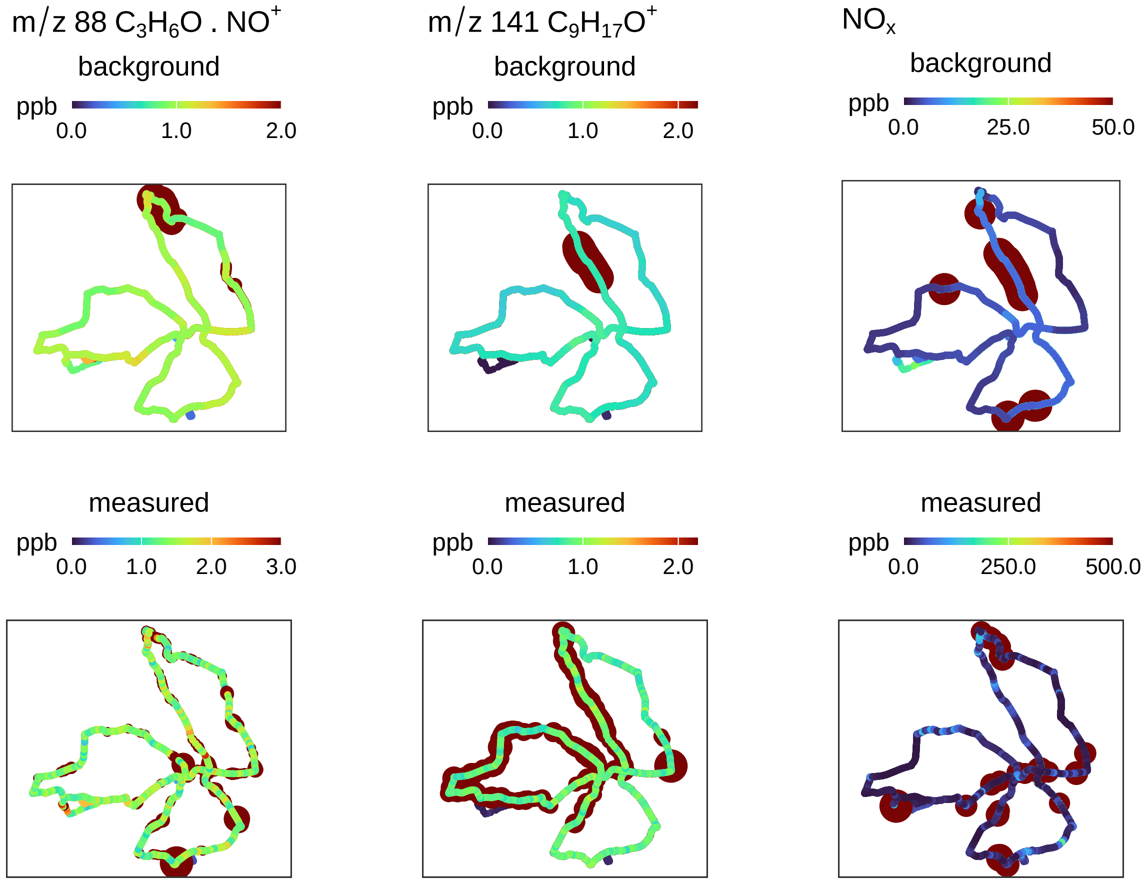

Figure S3: Spatial distribution of acetone, nonanal, and NO_x, representative tracers of personal care products, cooking, and traffic emissions. The top and bottom panels depict the background and measured concentration of the tracers from individual driving circuits, respectively. Background concentrations were calculated using the *rolling background* method. Larger marker sizes indicate values exceeding the scale.

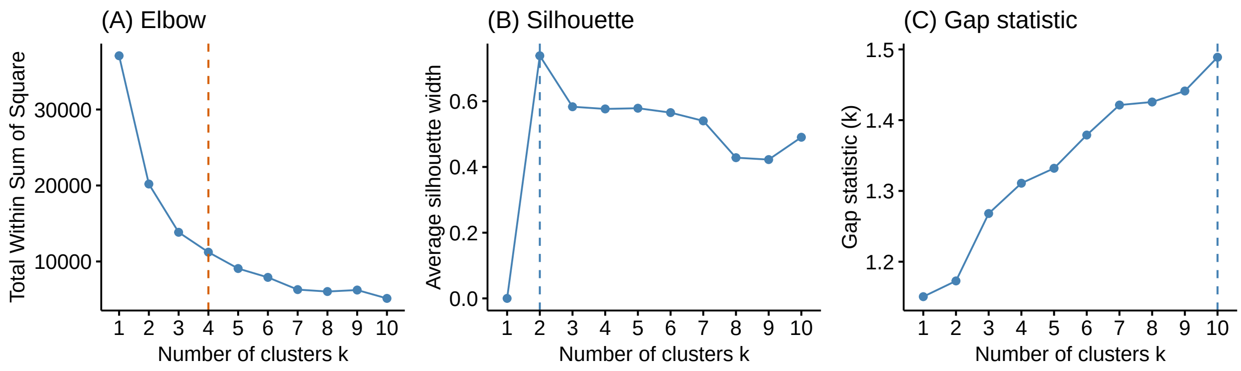

Figure S4: Three methods for determining the optimal K-Mean Clusters calculated for three types of emission sources. The dashed line indicates the suggested optimal number of clusters for each method. The orange dashed line in (A) represents the selected number of clusters. These clusters were used to analyze the clusters of sources and their relationship with the increment concentration of VOCs.

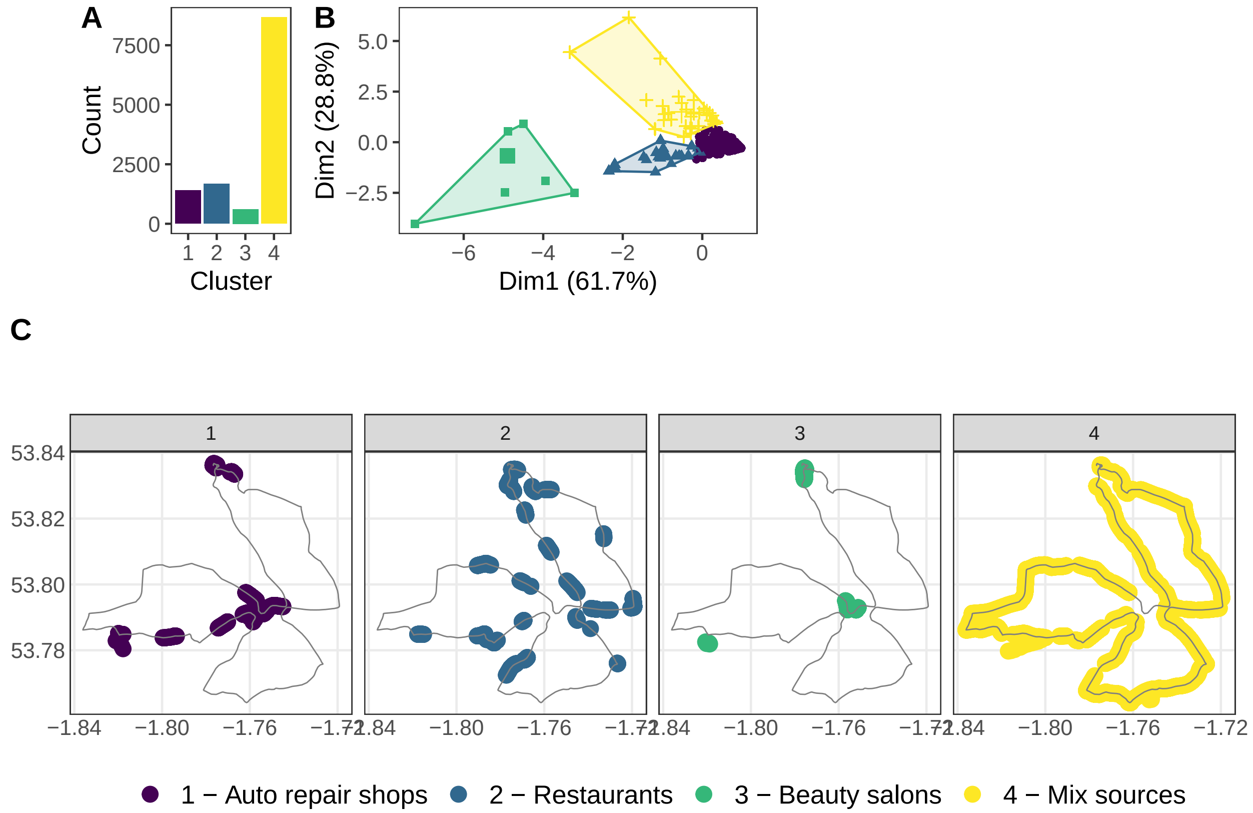

Figure S5: (A) Number of variables in each cluster. Each variable represents observation points from all measurement circuits, characterized by the sum of the weighting factor indicating source contribution. (B) Principal component analysis plot showing variables from each cluster, based on the first two principal components that capture the majority of the variance. (C) Spatial distribution of clusters mapped onto their corresponding locations along the measurement circuit.

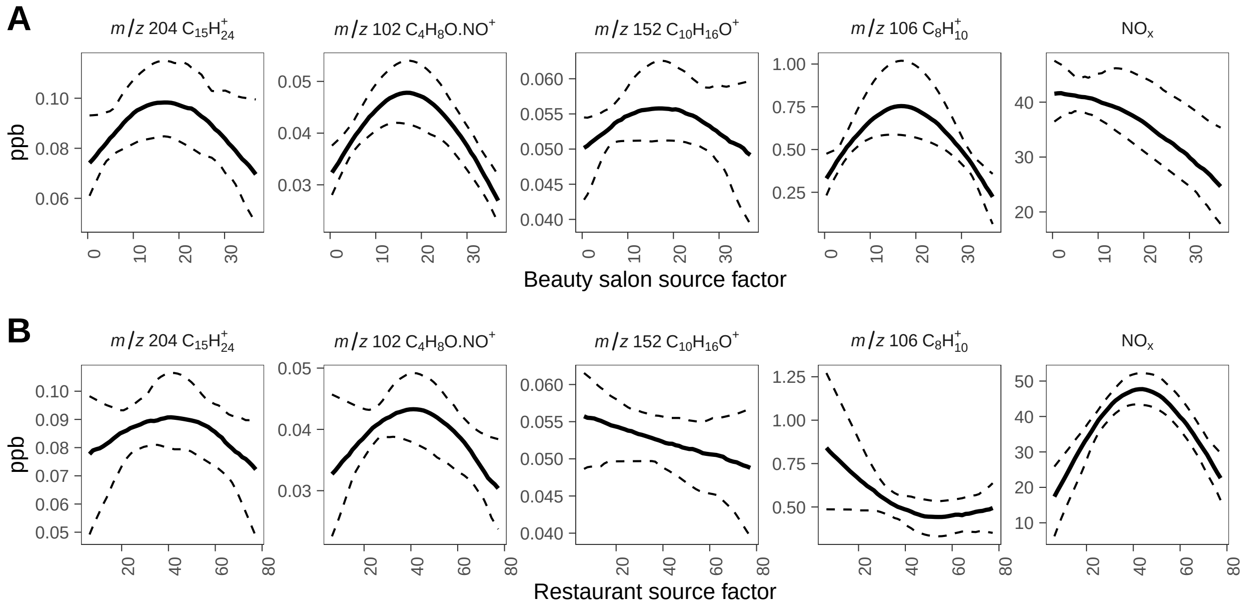

Figure S6: Additional GAM fits between *m/z* 102 ($C_4H_8O \cdot NO^+$; butanone), *m/z* 106 (${C_8H_{10}}^+$; C_2-alkylbenzenes), *m/z* 152 ($C_{10}H_{16}O^+$; citral), and *m/z* 204 (${C_{15}H_{24}}^+$; sesquiterpenes) and (A) beauty salon and (B) restaurant *source factors*. The lack of positive correlation indicates that these species do not co-vary with either *source factors*, suggesting minimal contribution from beauty salons or restaurants.

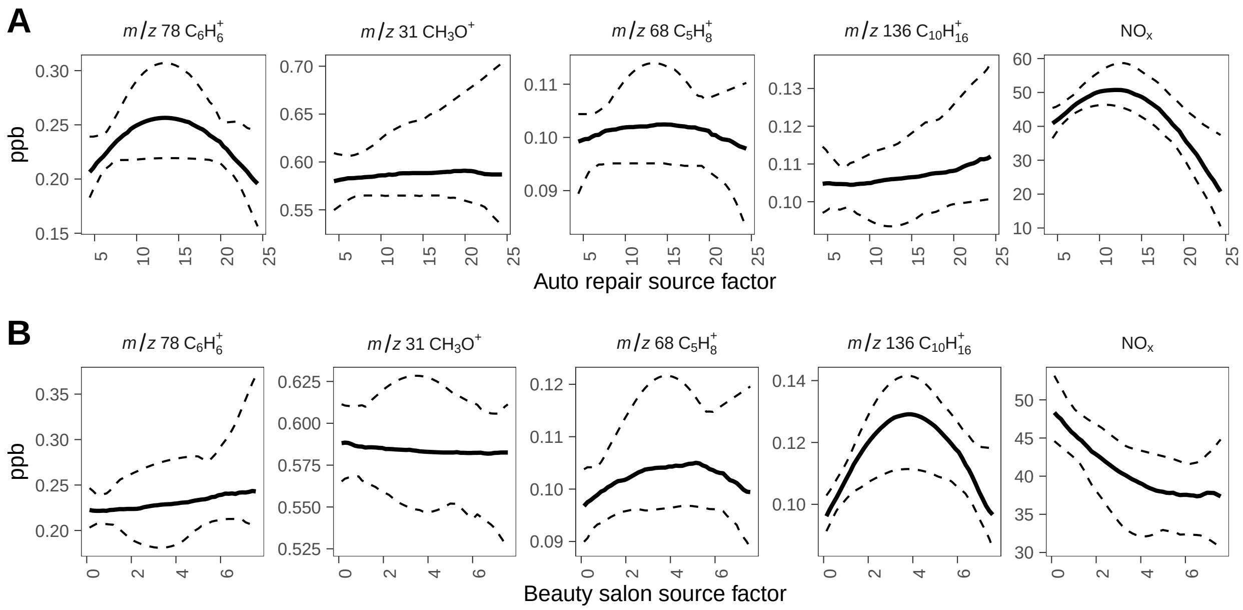

Figure S7: Additional GAM fits between *m/z* 31 (CH_3O^+; formaldehyde), *m/z* 68 (${C_5H_8}^+$; isoprene/furan), *m/z* 78 (${C_6H_6}^+$; benzene), and *m/z* 136 (${C_{10}H_{16}}^+$; monoterpenes) and (A) auto repair and (B) beauty salon shop *source factors*. The lack or absence of positive correlation and larger uncertainty indicate that these species do not co-vary with either *source factors*, suggesting minimal contribution from auto repair shops or beauty salons.

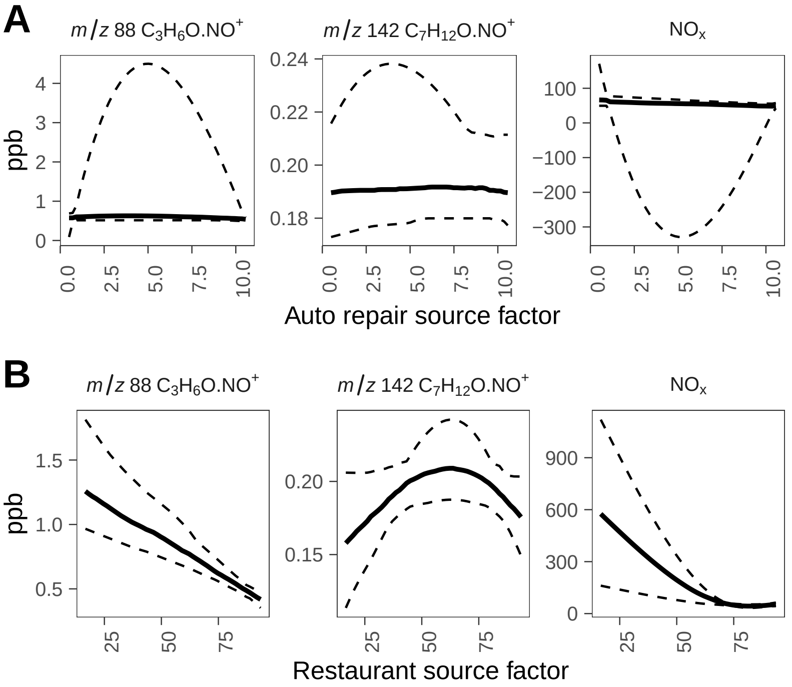

Figure S8: Additional GAM fits between *m/z* 99 ($C_3H_6O \cdot NO^+$; acetone) and *m/z* 142 ($C_7H_{12}O \cdot NO^+$; 2-heptenal) and (A) *auto repair* and (B) *restaurant source factors*. The absence of positive correlation and larger uncertainty indicates that these species do not co-vary with either *source factors*, suggesting minimal contribution from auto repair shops or restaurants.

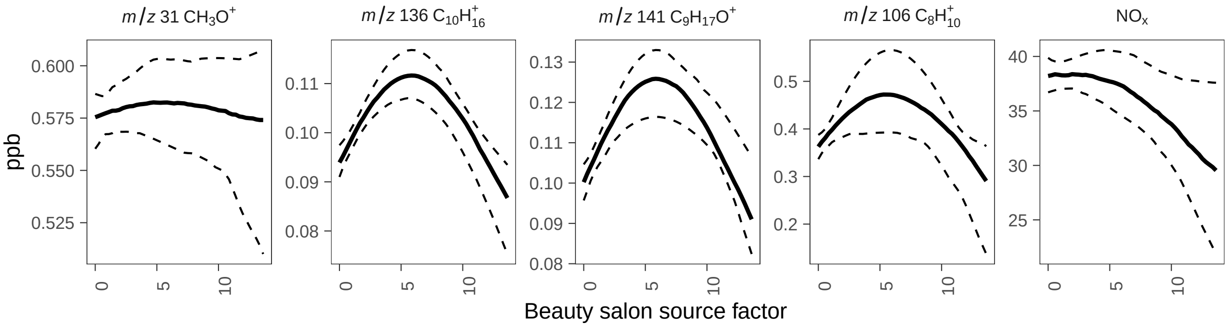

Figure S9: Additional GAM fits between *m/z* 31 (CH_3O^+; formaldehyde), *m/z* 136 (${C_{10}H_{16}}^+$; monoterpenes), *m/z* 141 ($C_9H_{17}O^+$; nonanal) and *m/z* 106 (${C_8H_{10}}^+$; C_2-alkylbenzenes) and the *beauty salon source factor*. The absence of correlations indicates no direct contribution from beauty salon activities to their concentration.

Table S2: Summary of tracer associations with published emission profiles.

**Source Type**	**VOC Tracer**	**m/z (Formula)**	**Reference(s)**	**Sources/mechanisms**
Auto Repair	Butanone	m/z 102 ($C_4H_8O.NO^+$)	[9]	Solvents and fuel components
	C2-alkylbenzenes	m/z 106 ($C_8H_{10}^+$)	[9–12]	Solvent, auto repair painting, vehicle exhaust, air freshener
	Citral	m/z 152 ($C_10H_{16}O^+$)	[13,14]	Essential oil in car disinfectant sprays
	Sesquiterpenes	m/z 204 ($C_15H_24^+$)	[13,15]	Essential oil, cleaning products
Restaurant	Formaldehyde	m/z 31 ($C_6H_6^+$)	[16,17]	Cooking
	Benzene	m/z 78 (CH_3O^+)	[16]	Cooking
	Isoprene/Furan	m/z 68 ($C_5H_8^+$)	[16,18]	Human breath during cooking preparation, thermal degradation of biomass/oils
	Monoterpenes	m/z 136 ($C_{10}H_{16}^+$)	[16,19]	Food ingredients, seasonings, and cleaning products
Beauty Salon	Acetone	m/z 59 ($C_3H_6O.H^+$)	[20–22]	Nail polish removers and cosmetic solvents
	2-heptenal	m/z 113 ($C_7H_{12}O.H^+$)	[23,24]	Skincare products
Mixed Source	C2-alkylbenzenes	m/z 106 ($C_8H_{10}^+$)	[9–12,16,25]	Solvent, auto repair painting, vehicle exhaust, car freshener, cooking, frying
	Formaldehyde	m/z 31 (CH_3O^+)	[9,12,16,17]	Car freshener, by-product of combustion, and cooking
	Monoterpenes	m/z 136 ($C_{10}H_{16}^+$)	[12,16,19]	Food ingredients, seasonings, and cleaning products, car freshener
	Nonanal	m/z 141 ($C_9H_{17}O^+$)	[16,25–27]	Cooking, frying, car interior materials

References

(1) Technologies, S. Quantitation: SIFT-MS Calibration Principles. *Syft Technologies Training Materials* **2014**,

(2) Langford, V. S.; Graves, I.; McEwan, M. J. Rapid monitoring of volatile organic compounds: a comparison between gas chromatography/mass spectrometry and selected ion flow tube mass spectrometry. *Rapid Communications in Mass Spectrometry* **2014**, *28*, 10–18, _eprint: https://analyticalsciencejournals.onlinelibrary.wiley.com/doi/pdf/10.1002/rcm.6747.

(3) Borge, R.; Jung, D.; Lejarraga, I.; de la Paz, D.; Cordero, J. M. Assessment of the Madrid region air quality zoning based on mesoscale modelling and k-means clustering. *Atmospheric Environment* **2022**, *287*, 119258.

(4) Dejamkhooy, A.; Dastfan, A.; Ahmadifard, A. K-means clustering and correlation coefficient based methods for detection of flicker sources in non-radial power system. *Russian Electrical Engineering* **2014**, *85*, 251–259.

(5) Kaufman, L.; Rousseeuw, P. J. *Finding Groups in Data*; John Wiley & Sons, Ltd, 1990; Section: 1 _eprint: https://onlinelibrary.wiley.com/doi/pdf/10.1002/9780470316801.ch1.

(6) Tibshirani, R.; Walther, G.; Hastie, T. Estimating the Number of Clusters in a Data Set via the Gap Statistic. *Journal of the Royal Statistical Society. Series B (Statistical Methodology)* **2001**, *63*, 411–423, Publisher: [Royal Statistical Society, Oxford University Press].

(7) Carslaw, D. C.; Beevers, S. D.; Tate, J. E. Modelling and assessing trends in traffic-related emissions using a generalised additive modelling approach. *Atmospheric Environment* **2007**, *41*, 5289–5299.

(8) Hastie, T. J. *Statistical Models in S*; Routledge, 1992; Num Pages: 59.

(9) Passant, N. R. *Speciation of UK emissions of non-methane volatile organic compounds*; 2002.

(10) Xiao, H.; Zhang, J.; Hou, Y.; Wang, Y.; Qiu, Y.; Chen, P.; Ye, D. Process-specified emission factors and characteristics of VOCs from the auto-repair painting industry. *Journal of Hazardous Materials* **2024**, *467*, 133666.

(11) Cliff, S. J.; Lewis, A. C.; Shaw, M. D.; Lee, J. D.; Flynn, M.; Andrews, S. J.; Hopkins, J. R.; Purvis, R. M.; Yeoman, A. M. Unreported VOC Emissions from Road Transport Including from Electric Vehicles. *Environmental Science & Technology* **2023**, *57*, 8026–8034, Publisher: American Chemical Society.

(12) Steinemann, A.; Nematollahi, N.; Weinberg, J. L.; Flattery, J.; Goodman, N.; Kolev, S. D. Volatile chemical emissions from car air fresheners. *Air Quality, Atmosphere & Health* **2020**, *13*, 1329–1334.

(13) Sharmeen, J. B.; Mahomoodally, F. M.; Zengin, G.; Maggi, F. Essential Oils as Natural Sources of Fragrance Compounds for Cosmetics and Cosmeceuticals. *Molecules* **2021**, *26*, 666, Number: 3 Publisher: Multidisciplinary Digital Publishing Institute.

(14) Jiang, J.; Ding, X.; Isaacson, K. P.; Tasoglou, A.; Huber, H.; Shah, A. D.; Jung, N.; Boor, B. E. Ethanol-based disinfectant sprays drive rapid changes in the chemical composition of indoor air in residential buildings. *Journal of Hazardous Materials Letters* **2021**, *2*, 100042.

(15) Harding-Smith, E.; Shaw, D. R.; Shaw, M.; Dillon, T. J.; Carslaw, N. Does green mean clean? Volatile organic emissions from regular versus green cleaning products. *Environmental Science: Processes & Impacts* **2024**, *26*, 436–450, Publisher: The Royal Society of Chemistry.

(16) Kumar, A.; O'Leary, C.; Winkless, R.; Thompson, M.; Davies, H. L.; Shaw, M.; Andrews, S. J.; Carslaw, N.; Dillon, T. J. Fingerprinting the emissions of volatile organic

compounds emitted from the cooking of oils, herbs, and spices. *Environmental Science: Processes & Impacts* **2024**, Publisher: The Royal Society of Chemistry.

(17) Ho, S. S. H.; Yu, J. Z.; Chu, K. W.; Yeung, L. L. Carbonyl Emissions from Commercial Cooking Sources in Hong Kong. *Journal of the Air & Waste Management Association* **2006**, *56*, 1091–1098, Publisher: Taylor & Francis _eprint: https://doi.org/10.1080/10473289.2006.10464532.

(18) Zhang, D.-C.; Liu, J.-J.; Jia, L.-Z.; Wang, P.; Han, X. Speciation of VOCs in the cooking fumes from five edible oils and their corresponding health risk assessments. *Atmospheric Environment* **2019**, *211*, 6–17.

(19) Arata, C.; Misztal, P. K.; Tian, Y.; Lunderberg, D. M.; Kristensen, K.; Novoselac, A.; Vance, M. E.; Farmer, D. K.; Nazaroff, W. W.; Goldstein, A. H. Volatile organic compound emissions during HOMEChem. *Indoor Air* **2021**, *31*, 2099–2117, _eprint: https://onlinelibrary.wiley.com/doi/pdf/10.1111/ina.12906.

(20) Lamplugh, A.; Harries, M.; Nguyen, A.; Montoya, L. D. VOC emissions from nail salon products and their effective removal using affordable adsorbents and synthetic jets. *Building and Environment* **2020**, *168*, 106499.

(21) Lamplugh, A.; Harries, M.; Xiang, F.; Trinh, J.; Hecobian, A.; Montoya, L. D. Occupational exposure to volatile organic compounds and health risks in Colorado nail salons. *Environmental Pollution* **2019**, *249*, 518–526.

(22) Tsigonia, A.; Lagoudi, A.; Chandrinou, S.; Linos, A.; Evlogias, N.; Alexopoulos, E. C. Indoor Air in Beauty Salons and Occupational Health Exposure of Cosmetologists to Chemical Substances. *International Journal of Environmental Research and Public Health* **2010**, *7*, 314–324, Number: 1 Publisher: Molecular Diversity Preservation International.

(23) Sun, L.; Wang, G.; Xiong, L.; Yang, Z.; Ma, Y.; Qi, Y.; Li, Y. Characterization of volatile organic compounds in walnut oil with various oxidation levels using olfactory analysis and HS-SPME-GC/MS. *Current Research in Food Science* **2024**, *9*, 100848.

(24) Poyato, C.; Thomsen, B. R.; Hermund, D. B.; Ansorena, D.; Astiasarán, I.; Jónsdóttir, R.; Kristinsson, H. G.; Jacobsen, C. Antioxidant effect of water and acetone extracts of Fucus vesiculosus on oxidative stability of skin care emulsions. *European Journal of Lipid Science and Technology* **2017**, *119*, 1600072, _eprint: https://onlinelibrary.wiley.com/doi/pdf/10.1002/ejlt.201600072.

(25) Schauer, J. J.; Kleeman, M. J.; Cass, G. R.; Simoneit, B. R. T. Measurement of Emissions from Air Pollution Sources. 4. C1-C27 Organic Compounds from Cooking with Seed Oils. *Environmental Science & Technology* **2002**, *36*, 567–575, Publisher: American Chemical Society.

(26) Tokumura, M.; Hatayama, R.; Tatsu, K.; Naito, T.; Takeda, T.; Raknuzzaman, M.; Habibullah-Al-Mamun, M.; Masunaga, S.; Tokumura, M.; Hatayama, R.; Tatsu, K.; Naito, T.; Takeda, T.; Raknuzzaman, M.; Habibullah-Al-Mamun, M.; Masunaga, S. Car indoor air pollution by volatile organic compounds and aldehydes in Japan. *AIMS Environmental Science* **2016**, *3*, 362–381, Cc_license_type: cc_by Primary_atype: AIMS Environmental Science Subject_term: Research article Subject_term_id: Research article.

(27) Buchecker, F.; Loos, H. M.; Buettner, A. Smells like new car or rather like an old carriage? - Resolution of the decay behavior of odorants in vehicle cabins during usage. *Indoor Air* **2022**, *32*, e13112, _eprint: https://onlinelibrary.wiley.com/doi/pdf/10.1111/ina.13112.
